# Default Reference Frames for Angular Expansion in the Perception of Visual Direction

**DOI:** 10.3390/vision8010007

**Published:** 2024-02-21

**Authors:** Prince U. D. Tardeh, Crystal Xu, Frank H. Durgin

**Affiliations:** Department of Psychology, Swarthmore College, 500 College Ave., Swarthmore, PA 19081, USA; ptardeh1@swarthmore.edu (P.U.D.T.); crystalmxu@gmail.com (C.X.)

**Keywords:** horizon, angular declination, intrinsic bias

## Abstract

Prior work has shown that perceived angular elevation relative to a visible horizon/ground plane is exaggerated with a gain of about 1.5. Here, we investigated whether estimates of angular elevation remain exaggerated when no such visual gravitational reference is provided. This was investigated using a series of five experiments, with most using a novel apparatus to view a large field-of-view stereoscopic virtual environment while lying supine, looking straight up. Magnitude estimation methods were used as well as psychometric matches to internal standards with a total of 133 human participants. Generally, it was found that the exaggerated scaling of elevation seemed to be a default for 3D space, even if testing was performed in virtual environments that were nearly empty. Indeed, for supine observers, a strong exaggeration was found even for azimuthal judgments, which is consistent with the idea that, when looking upward, all deviations are in elevation. This suggests that the overarching gravitational frame often serves as a default reference frame.

## 1. Introduction

For the case of a standing observer, students of space perception are aware of several distortions in locomotor space (the scales of space in which locomotor action takes place). Ground distances appear shorter than they should [1,2,3,4,5,6,7,8,9,10,11]; hills appear steeper than they are (both uphill and downhill) [12,13,14,15,16,17,18,19,20]; and large (frontal) vertical extents appear much larger than corresponding horizontal frontal extents [21,22,23,24,25]. According to the angular expansion hypothesis, these effects are related to an efficient coding strategy for angular variables that densely represents the most useful angular information [26,27]. For example, the direction of gaze in the sagittal plane (sometimes called the angle of regard, or angular declination) is an excellent source of information about ground distance that can effectively control action [28,29,30,31,32,33,34], but it is typically used for ground distances of more than two strides, meaning that most of the time, we are concerned with discriminating among visual directions in the sagittal plane that are less than 45° from the visual direction that is straight ahead [35,36,37,38].

Convergent measures have shown that the perceived visual direction in the sagittal plane (angular elevation) is systematically misperceived by a factor of about 1.5, as established by explicit, implicit, and non-verbal action measures both in the real world and in virtual environments [22,26,39,40], and in haptic perception as well as visual perception [41]. This magnitude of angular misperception quantitatively predicts the typical underestimation of perceived distance along the ground [26] and is still present even in participants whose explicit knowledge of the ground distance in familiar units is cognitively calibrated [42]. These angular distortions are thought to be undetectable in many action measures (such as walking to a previewed target without vision) that have been proposed to show accurate perception [29,43,44] because action is already calibrated to the experienced outcomes of commonly engaged action, such as walking [45]. Similar angular expansion ideas have been applied to hill perception, which has been used to establish refinements of the model [46]. Thus, the angular expansion hypothesis provides a unifying theoretical basis for several dramatic spatial biases that have been reported for locomotor space.

Although the geometry of perceived hill slant and ground distance both typically involve a metric misperception of world geometry in the sagittal plane involving perceived angular elevation and slant, there are also misperceptions of the extent of visual direction to the left and right of the visual direction that is straight ahead (perceived azimuth) [47]. Note that we are not referring to visible angles that are perceived in perspective [48], but to the perceived (egocentric) visual direction itself. Distortions in the perceived azimuth direction tend to be somewhat smaller (e.g., a gain of about 1.2) than those in elevation [47,49,50].

In the classic horizontal/vertical illusion (HVI), small vertical lines appear about 5% longer than equivalent horizontal lines, and the effect is yoked to head orientation [51,52,53,54]. But the much larger angular distortions in azimuth and elevation have been shown to be encoded in ground-based coordinates, such that they are maintained even by observers looking at the real world on their side [23,47] (unless they are positioned quite near the ground [55]). In a particularly dramatic demonstration conducted in a large outdoor space, it was shown that the large-scale HVI [55,56,57], which has a magnitude of about 25%, can be decomposed into a 5% illusion that is retinotopic (the classic HVI) and a 20% illusion that is allocentric, or ground-based, exactly corresponding to the ratio of the 1.5 angular gain in elevation and the 1.2 angular gain in azimuth which were observed in studies investigating the scale expansion hypothesis [23,26]. Another study has shown that these underlying angular distortions can be masked for size judgments in near space when alternative perceptual strategies are available, such as retinal size comparison, but they re-emerge even for relatively short objects that are separated in depth [58]. The angular expansion of elevation can also be artificially augmented by interference from other perceptual dimensions, such as the ground distance to the straight-ahead reference [59]. Both in the real world and in virtual environments, they tend to survive changes in viewer orientation [23,47], but they are carried along with 90° rotations of the visual world in head-mounted displays [23].

The current investigation was inspired by a prior work examining the role of the perceived ground plane on the explicit estimation of the perception of the azimuth. Using a large stereoscopic virtual display, it was found that, for a seated observer, estimates of the visual direction to a depicted ball off to one side (relative to a distant reference post straight ahead) were expanded with a gain of 1.25 when the scene depicted a ground plane, but they were only exaggerated with a gain of about 1.05 when a world was not depicted, other than the central vertical line extending from the bottom to the top of the screen [60]. In other words, the perceptual expansion of the angular direction in azimuth seemed to be triggered by the ground plane being visible. The present investigation sought to examine whether or not the expansion of perceived elevation was likewise dependent on the presence and orientation of a ground plane.

One reason to suspect that the exaggeration of elevation might be less likely to switch off, as it were, is that our typical range of environmental examination is quite limited in angular elevation. In normal life, we turn our bodies in azimuth as we navigate, and we can quickly scan quite far left and right, but we rarely look directly up or directly down as we evaluate our environment. We can look up at tall nearby objects, but the experience can be almost disorienting at times. When looking straight down, the ground becomes a near frontal plane. A previous study that removed the visible ground plane but left a horizon line visible found a typical angular expansion [59], but in that study, the participants were seated upright, so it is easy to imagine that the gravitational reference frame was strongly supported by that alone. The present investigation primarily uses supine observers looking upward in an attempt to remove the normal gravitational reference frame and avoids providing any horizon in several experiments. In addition, the investigation introduces inverted (upside down) visual environments to supine observers to see whether this inversion affected the perceived space in any way, as measured by reports of apparent angular elevation.

Three experimental studies are reported here. In the first study, we sought to measure the perceived direction in elevation for supine observers looking up along the direction of gravity. This was carried out to try to avoid the imposition of a gravitational reference frame from gravity itself, and we contrasted three different kinds of visual environments (VE), including a world consisting solely of a straight-ahead reference ball, and a test ball that was positioned at various directions in the sagittal plane defined by the observer’s body. The other two VEs included ground planes in addition to the two balls. One was oriented normally (upright)—as if the supine observer was standing on the ground (with an eye height of 1.6 m). The other VE was oriented upside down (inverted)—mimicking the upright world in all respects except orientation. Within this first study, three distinct experiments were run, which will be labeled 1A, 1B, and 1C.

The second study reverted to comparing perception in empty and ground-plane VEs with a seated upright observer. This experiment was undertaken to test whether the results found for empty VEs with supine observers (which, to anticipate, showed elevation gains of about 1.5) could be generalized to upright observers. Additionally, this second study introduced a non-numerical measure to estimate the angular gain.

Finally, in a third study, we returned to the case of the supine observer, using the non-numerical measure to compare judgments of azimuth and elevation (relative to the observer) that were made while in a supine position in the presence or absence of an appropriately oriented ground plane in the VE. The three studies, taken together, will tend to suggest that angular expansion in perceived elevation is fairly robust even in nearly empty VEs, even if the primary reference frame is intended to be the observer’s own body. There was evidence of some forms of modulation of the perception of elevation by environmental factors.

## 2. Materials and Methods

The three studies (comprising 5 experiments) presented here were conducted using two different back-projection screens that preserved polarization for stereoscopic projection; participants wore custom 3D polarizing glasses specifically designed to allow them to view large visual angles along the angular direction to be measured, and the interocular distance of each participant was measured and used to render the virtual environment with the intended retinal disparities. The projector was a ProPIXX with an active circular polarizer for alternating frame projection at 120 Hz and a resolution of 1920 × 1080 pixels. The VR software used for projection was Vizard 5.0. The methods of measurement, apparatuses, virtual environments used, and procedures are described below.

The studies reported here were conducted in accordance with the Declaration of Helsinki, and they were approved by the Swarthmore College IRB, protocol number IRB-FY22-23-14. Written informed consent was obtained from all participants. All participants were naïve to the goals of the experiment in which they participated, and they were either paid for their time or participated as part of a research participation requirement for an Introduction to Psychology course. Experiments 2 and 3 were pre-registered at AsPredicted.org (Experiment 2: https://aspredicted.org/fe9mh.pdf (accessed on 15 February 2024); Experiment 3: https://aspredicted.org/mr9vy.pdf (accessed on 15 February 2024)). Data files and analysis files for all experiments are available at OSF (https://osf.io/a2xue/?view_only=08e3dfaafcde47a0b930d33d800327bc (accessed on 15 February 2024)).

### 2.1. Methods of Measurement

Two different measurement methods were used in the present experiments.

#### 2.1.1. Magnitude Estimation

For Experiments 1A, 1B, 1C, and phase 2 of Experiment 2, participants made verbal estimates of the perceived visual direction of the target ball relative to the straight-ahead reference ball. They orally reported the magnitude of the deviation in degrees without regard to sign. These oral estimates were typed into the computer by the experimenter. In Experiment 2, where the experimenter sat in a different room from the participant, some of these estimates were later corrected by two authors who considered only cases of clear outliers where there was a clear possibility of auditory confusion (e.g., “seventy” instead of “seventeen” and vice versa). A typical estimation procedure entailed a series of 3 blocks of judgments for each of the 11 angles, ranging from 6° of elevation to 36° of elevation by 3s (or −6° to −36°) in random order for each tested condition (usually two conditions per participant). Of theoretical interest were the slopes of the lines fitting these estimates (separately for each condition) to see if they resembled or departed from 1.5.

#### 2.1.2. Psychophysical Judgments with Respect to an Internal Standard

For Experiment 3 and for the first phase of Experiment 2, participants simply had to judge on each trial whether the presented target was more or less than a verbally specified target angle. This target angle was 30° in Experiment 2, and there were 52 test trials based on 4 interleaved staircases starting at 15, 25, 35, and 45° (step size 8°; outer bounds of 2° and 52°). The verbally specified target angle in Experiment 3 was 45°, which was described as halfway between straight ahead and either straight up for elevation, or directly to the left for azimuth (all described relative to the head); 60 judgments were determined using an interleaved staircase method with initial values that started at 10, 22, 34, 46, and 58° from straight ahead (step size: 10°; outer bounds of 10° and 66°). Cumulative normal psychometric functions were fit to these judgments.

### 2.2. Apparatuses

**Figure 1:**
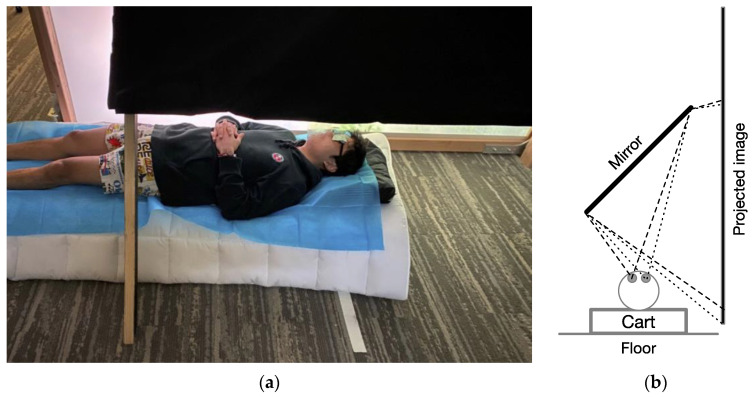
(**a**) Photograph of an apparatus for supine viewing of back-projected VE looking up through a mirror angled at 45° to a vertical projection screen. Open-frame glasses with large, flexible, circularly polarized filters allowed for a large, stereoscopic vertical field of view. (**b**) Schematic diagram of optical projection system that produces a virtual image directly above the viewer.

In Experiments 1A, 1B, 1C, and 3, a horizontally oriented back-projection screen was used, with a large mirror (0.58 × 1.62 m) mounted at 45° which was positioned so that participants could lie supine on a very low table that could be rolled under the mirror to view the projected image as if it were directly above them. Figure 1 shows an image of a model demonstrating the apparatus, as well as a schematic of the viewing geometry. The projected image area was 2.56 × 1.44 m (1920 × 1080 pixels) on the projection screen, though the narrower dimension visible through the mirror system was 1.27 m. The perpendicular viewing distance to the center of the view on the screen through the mirror was 0.83 m.

The position and orientation of the viewer relative to the display varied based on condition. For Experiments 1A–1C, where visual elevation both upward and downward relative to the observer were assessed, supine participants were positioned parallel to the long axis of the mirror with their eyes at the center of the visible display so that the boundaries of visual elevation available for rendering ranged from −57° to +57°. The field of view was 75° wide. For Experiment 3, participants were positioned under the mirror parallel to the screen if they were judging elevation; they were positioned perpendicular to the screen if they were judging azimuth. In both cases, their viewing positions were offset by 55 cm along the long axis of the display, such that the maximum visible direction along the tested direction (from their midline) was 66°.

In Experiment 2, a different apparatus was used to test upright observers. Specifically, a tall (vertically oriented) back-projection screen was used with the observer sitting upright in an adjustable-height chair with their head in chin- and forehead rests that were used to standardize the viewing eye height and distance. For this apparatus, the perpendicular viewing distance to the screen was 0.93 m. The projected display for this experiment was 1.24 m wide and extended 1.13 m upward and 1.10 m downward from the seated eye height of each observer, meaning that the maximum visible elevation angle both upward and downward was about 50°, and the horizontal field of view was 67°. This large vertical extent was achieved by turning the projector on its side.

A limitation of all of these apparatuses is that they do not entirely hide physical surroundings. Although room lights were turned off during the experiment, ambient light from the projected image was available to dimly illuminate the surroundings.

### 2.3. Virtual Environments

**Figure 2:**
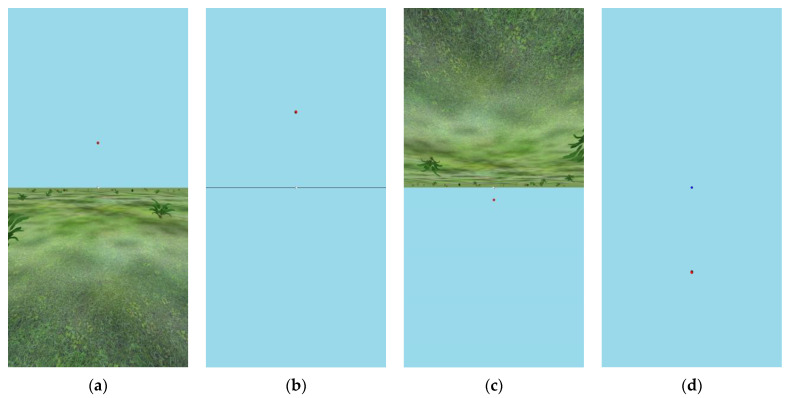
(**a**) Upright virtual environment (VE) used in Experiment 1A, (**b**) empty VE used in Experiment 1A, (**c**) inverted VEs used in Experiments 1A and 1B; in Experiment 1B, the red ball could be presented toward the ground as well as toward the sky. (**d**) The sky-only VE used in Experiment 1B. Actual displays included disparity information to specify depth. Foreground plants were richly structured in 3D. Accurate 3D perspective would require viewing each image from a distance of 1/3 its height from directly in front of its center. The images shown are screen captures that have been cropped horizontally to show only the portion that was visible through the mirror system.

The virtual environments (VEs) used were fairly similar across all five experiments. All of the VEs were similar to the ones used previously in our lab to represent ground planes in a related study [59] but with two alterations. First, in all experiments but Experiment 1C, the two balls—one straight ahead, and one whose direction was to be judged—were presented at a simulated distance of 1 m from the observer. This near distance was used because pilot testing had shown that when a background texture was not present, many pilot subjects reported diplopia for farther balls, indicating that they were unable to fuse the two eye images for farther distances (probably due to conflict between vergence and accommodation information) without the supporting disparity gradient of the ground plane. Second, whereas the older displays used large objects (trees) in the background to aid with stereoscopic depth, all of the present studies, except Experiment 1C, sought to avoid features that protruded into the sky. To this end, the distant features were rendered shorter than eye height to avoid such protrusion. Eye height was simulated as 1.6 m in all experiments but Experiment 2, where it coincided with the floor level in the room, 1.19 m. Even low objects aid binocular registration to support the perception of a ground plane. Figure 2 shows the VEs used in Experiments 1A and 1B. For Experiment 3, where azimuthal judgments were studied, all distant features were removed, leaving only the grass-like ground.

### 2.4. Experimental Designs

#### 2.4.1. Experiments 1A, 1B, and 1C: Elevation Estimates with Supine Observers

In Experiment 1A, there were 25 college student participants, randomly assigned to conditions. All participants always saw the red target balls in an untextured “sky” portion of the VE. In counterbalanced order, they made judgements of balls that were both above (33 trials) and below (33 trials) them, with (also in counterbalanced order) either the ground portrayed (always in the non-judged region) or without a ground portrayed. If a participant started with the ground present, and the world upright, for example, they would see balls in their upper visual field for 33 trials (3 blocks of each of 11 angles ranging from 6 to 33 degrees by 3 s, presented in random order), and after a short break, they would then be placed in a VE that did not have a ground plane, but only a thin black horizontal line and a single white ball straight ahead, and the red test ball would always appear in the lower part of their visual field in the second half of the procedure for them. Each participant always made judgments about a ball presented against a blank blue portion of the VE. When the ground plane was present, 14 of the participants saw an upright world, and the other 11 saw an upside-down world.

The balls in all experiments but Experiment 1C were each simulated as 2 cm in diameter, and thus subtended a visual angle of 1.15° at the 1 m simulated viewing distance. A red target ball and a white (light gray) reference ball were used in Experiment 1A, but the reference ball was made dark blue in Experiment 1B against the empty sky, and the target ball was white (light gray) and the reference ball was black (dark gray) in the remaining experiments, which were changes that were made to match prior studies [59].

For Experiment 1B, 20 (different) college undergraduates were assigned to conditions by alternation. In this version, the presence of a ground plane was manipulated between participants. That is, participants saw either an upside-down world throughout the experiment (like the upside-down world in Experiment 1A) or an empty world (all blue background), as shown in panels c and d in Figure 2. In this experiment, upward and downward angular deviations were randomly intermixed. This experiment, in which there was no horizon in the control VE, allowed for the clearest analysis of how judgments of elevation are impacted (or not) by not having a ground plane at all (for the 10 participants in that condition) or by having an upside-down perspective (for the other 10 participants).

For Experiment 1C, 22 (different) college students were each assigned to one of two conditions through alternation. The data of 2 participants (both in the upright world condition) were eliminated due to a lack of correlation between the responses and the presented stimuli, leaving 20 participants available for analysis. The goal of the experiment was to examine the effects of world orientation only, so the ground plane was always present, and a richer ground layout and a textured sky were used to better support a gradient of depth perception. In addition, the balls were simulated to lie either on the ground plane (with a concomitant change in retinal size according to viewing distance – following a method used in a previous publication [59]) or, if in the visual sky, at a constant height above the ground, receding in distance in the same way as they did along the ground as angular deviation decreased. In this experiment, there was no condition in which the ground plane was entirely removed. The main purpose of conducting this experiment was to test for any possible effect of world orientation with as rich a VE as possible. In a previous study, with upright observers, the use of a distant reference point produced an elevated intercept, which was the focus of the earlier study [59]. That study showed that the effect seemed to depend on the conflation of a large distance in depth between the reference and test ball with angular distance (observed both in VEs and real outdoor environments). To determine whether this pattern persisted (a) with supine observers, (b) with inverted environments, and (c) with balls presented in the sky, the straight-ahead reference ball was simulated as 40 cm in diameter at a distance of 50 m (0.46° visual angle) for the present experiment only, and the test ball was simulated as 10 cm in diameter, and was shown at simulated distances ranging from 2.7 m (2.2 m along a plane 1.6 m from eye level) to 15.3 m (15.2 m along a plane 1.6 m from eye level). Each participant saw either an upright world or an upside-down world only. Each participant made judgments that were blocked by whether the deviation was toward the sky or the ground. The ball colors used here were light gray for the test ball and dark gray for the reference ball, replicating the previously published experiment with upright observers [59].

The main design differences between Experiments 1A, 1B, and 1C, as well as the magnitude estimation portion of Experiment 2, are summarized in Table 1. In all of the initial experiments (1A, 1B, 1C), the participant lay supine, and their task was to orally provide numeric estimates in degrees of angular deviation in elevation from straight ahead. In Experiment 2, the same task was carried out while sitting upright. Table 2 summarizes the designs of the psychometric methods used with internal standards in phase 1 of Experiment 2 and in Experiment 3.

#### 2.4.2. Experiment 2: Elevation Perception with Upright Observers

**Figure 3:**
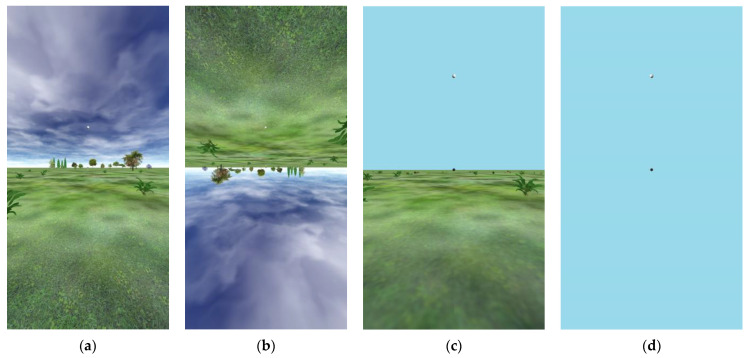
(**a**) Upright VE used in Experiment 1C. (**b**) Inverted VE used in Experiment 1C. (**c**) Upright VE used in Experiment 2. (**d**) Only sky VE used in Experiment 2. Upward elevations (relative to the observer) are shown here, but in all of these VEs, both upward and downward elevations were tested.

Experiment 2 was conducted with 24 college students assigned to a condition through alternation. Each participant was seated upright in front of a vertical screen that showed (for half the participants) (a) a VE with a normal ground plane at the height of the floor of the room in which they sat with a featureless blue sky, or (for the other half of the participants) (b) a VE that consisted of only a featureless blue sky and the two balls. These two VEs are shown in panels c and d in Figure 3. In both VEs, there was a dark reference ball, straight ahead, and a light test ball at some elevation above or below the reference ball.

The phase 1 task for each participant was to judge, on each trial, whether the visual direction of the test ball was higher or lower than 30° of upward elevation. There were 52 trials in this task, all using an upward elevation for the test ball. The screen appeared blank gray (fog was simulated) for a few seconds between trials to conceal the repositioning of the ball. This phase allowed us to compare the perceived 30° across the two VEs.

In phase 2, the participants remained in the same environment as in phase 1, but gave verbal estimates of elevation. Because all judgments in phase 1 were skyward, in the first block of 33 trials in phase 2, estimates were made for test balls shown in directions that were below the direction that was straight ahead, and in a second block of 33 trials, estimates were made for test balls that were above the direction that was straight ahead.

#### 2.4.3. Experiment 3: Comparing Azimuth with Elevation Perception in Supine Observers

**Figure 4:**
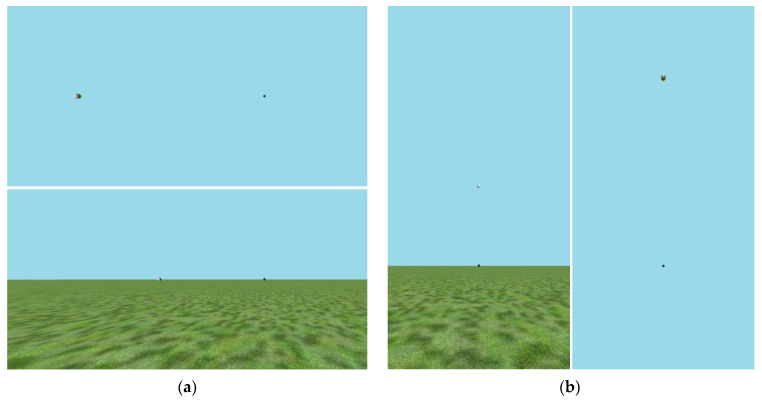
The four virtual environments used in Experiment 3 from the perspective of the supine viewer directly looking up at the small black ball. Note that the light and dark balls appeared to be the same size to the participants. Note also that the lighter ball appears both larger and elongated in the images showing large eccentricities here because it would have been optically farther from the viewer, and it would have been viewed obliquely (such that it would be circular in the retinal projection), because it represents a shaded sphere at the same radial distance, as specified by binocular disparity information in the actual display. (**a**) The VE with only the sky (top) and the VE with ground plane (bottom) for judgments in azimuth. (**b**) The ground-plane VE (left) and VE with only the sky (right) used for judgments in elevation.

Experiment 3 was conducted with 42 college student participants, each lying supine under the mirror apparatus. Whereas the previous experiments all examined the effects of visual environment on judgments of elevation, this experiment sought to directly contrast how judgments of elevation and azimuth might be affected by the presence of a ground plane. Consequently, participants were assigned, through alternation, to one of four different testing conditions which crossed (a) whether or not a ground plane was present and (b) whether they were judging elevation or azimuth. In all cases, the task was to judge whether the ball was more or less than 45° from the visual direction that was straight ahead. Trial images from all four VEs are shown in Figure 4 with the image orientation as it would have been from the observer’s vantage point. Note that the ground plane was relatively featureless to avoid providing landmarks that might affect azimuth judgments. The viewer was centered on the part of the image showing the small black ball to allow for a greater angular range either in azimuth (to the left of the viewer) or in elevation (above the viewer).

## 3. Results

To facilitate a concise presentation, the results of each experiment will be presented and discussed according to the four main empirical questions to be addressed:

1. Does the presence/absence of a ground plane affect the perception of the angular elevation? (Experiments 1A, 1B, 2, and 3.)
2. Does the apparent orientation of the environment affect the perception of the angular elevation? (Experiments 1A, 1B, 1C.)
3. Does the orientation of the observer (supine or upright) affect the perception of the angular elevation? (Experiment 2.)
4. Does the perception of the body-defined azimuth behave differently from the perception of the body-defined elevation for supine observers? (Experiment 3.)

### 3.1. Results of Experiments 1A and 1B: Strong Angular Expansion in the Absence of a Ground Plane

**Figure 5:**
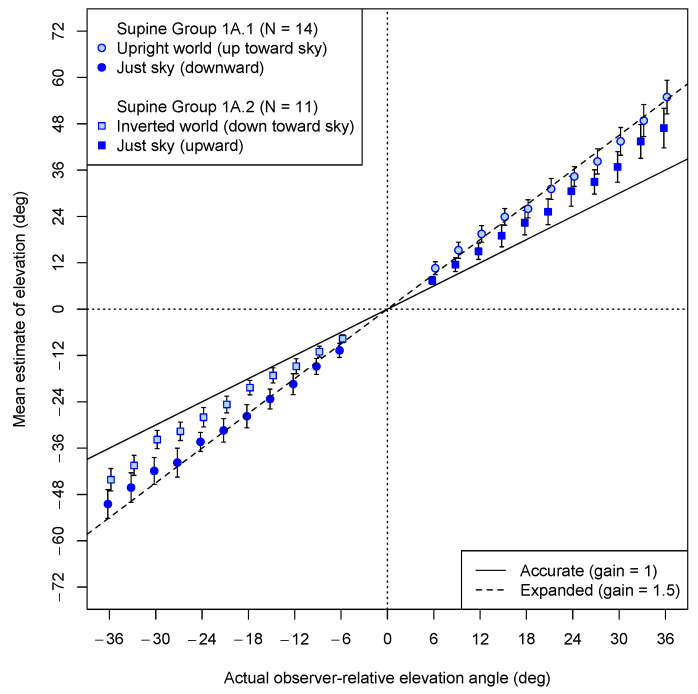
Experiment 1A results: Mean estimates of angular elevation in blue sky portion of VEs. Light blue symbols represent estimates given for balls in the sky, when a ground plane was present in the VE (upside down for estimates of negative (downward) angles, and right side up for estimates of positive angles). Darker blue symbols represent estimates made in the absence of any ground in the VE. Note that symbols of the same shape represent judgments made by the same participants. The dashed line shows the prediction of angular expansion to be 1.5. The solid line shows where accurate estimates should fall. Error bars represent standard errors of the means. Plot points have been offset horizontally by ±0.2 degrees to avoid overlap of error bars.

In Experiment 1A, elevation estimates were made by supine observers exclusively for balls that were presented against an empty blue background, either in a world not containing any other structure or with an upright or inverted ground plane presented in the untested portion of the visual field. Figure 5 shows the mean estimates for each elevation angle tested (with downward angles represented as negative numbers). The angular gains are defined by the slopes of best fitting lines computed separately for positive and negative angles for each participant. The angular gains were marginally lower when the VE was visibly upside down (M = 1.19; 95% CI: 1.00, 1.38) than when the VE was normal—ground below; sky above—(M = 1.48; 95% CI: 1.23, 1.74), (Welch’s *t*(22.1) = 2.03, *p* = 0.054). In contrast, the gains in the conditions without any ground at all (overall M = 1.37; 95% CI: 1.18, 1.56) appeared intermediate, but they did not differ reliably from 1.5 (*t*(24) = 1.46, *p* = 0.16). However, because each participant in Experiment 1A saw both a world with a ground plane and a world without a ground plane, it may be that judgments from one view carried over to the other, producing the apparent null difference between an upright world and no world at all. A stronger between-subject test was made in Experiment 1B of whether the presence of a ground plane mattered, while the issue of whether the world orientation had a consistent effect was investigated in Experiment 1C.

**Figure 6:**
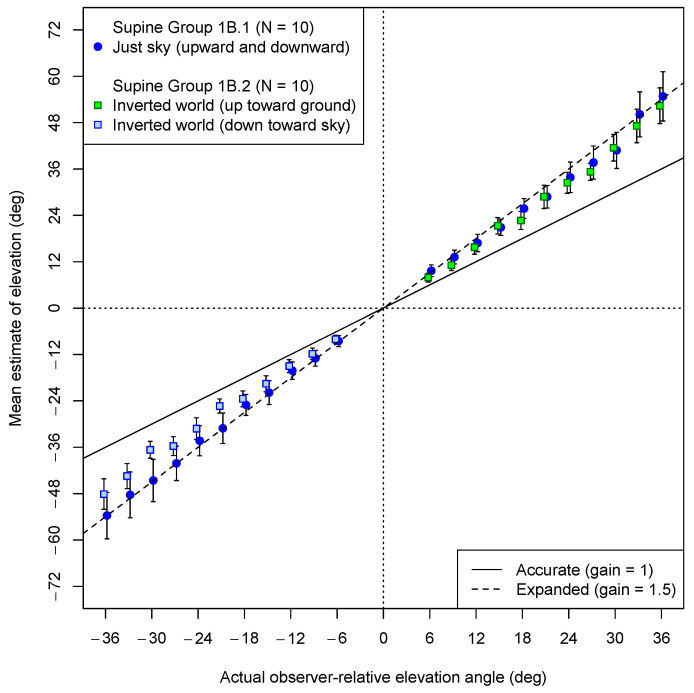
Experiment 1B results: Mean estimates of angular elevation both upward (positive) and downward (negative) one of two VEs (up and down judgments were intermixed). Square symbols represent estimates made by participants with an inverted ground plane in their VE, and the colors of the square plot points indicate whether the test ball was against an empty sky (blue) or the ground (green). Blue circular symbols represent estimates given by participants for whom the VE was just sky. An upside-down ground plane VE was present during estimation (up and down judgments were intermixed). The dashed line shows the prediction of angular expansion by 1.5. The solid line shows where accurate estimates would have fallen. Error bars represent standard errors of the means. Plot points have been offset horizontally by ±0.2 degrees to avoid overlap of error bars.

In Experiment 1B, with the ground plane upside down whenever it was present, the participants made estimates both upward (toward the inverted ground) and downward (toward the inverted sky). Whether a ground plane was visible or not was manipulated between participants. Both for the participants who saw a ground plane (albeit inverted) and for those tested without a ground plane in the VE, the up and down judgments were randomly intermixed. The average estimates are shown in Figure 6. The data from participants who saw the (upside down) ground plane are marked by squares. The data of those who saw an empty blue sky in the VE are indicated by dark blue circles. As is evident in the figure, both groups showed strong angular expansion when looking up. Indeed, the mean gain for those observing only a sky was almost exactly 1.5 both when looking up (M = 1.48; 95% CI: 1.03, 1.92) and when looking down (M = 1.51; 95% CI: 1.06, 1.96). A similar gain was observed when looking up toward the inverted ground plane (1.46; 95% CI: 1.03, 1.92). However, as in Experiment 1a, the estimates for the downward elevations (toward the “sky”) when an upside-down ground plane was shown (M = 1.31; 95% CI: 1.10, 1.52) seemed to show less expansion. In this case, the mean gain was again marginally less than 1.5 (*t*(9) = 2.06, *p* = 0.07). The replication of the exceptional condition observed in Experiment 1a is notable, even if the source of this reduced bias is unclear.

Most clearly, however, with regard to the supine observers, these results confirm that the presence of a visual ground plane is not necessary to observe angular expansion. Even if only a single reference ball was shown with neither a ground plane nor even a horizon line, the estimates of elevation were exaggerated by a factor of about 1.5. It is less clear why there seems to be less angular expansion reflected in the estimates for the balls in the “sky” when an inverted ground plane is visible in the upper visual field.

### 3.2. Results of Experiment 1C: Strong Angular Expansion Even with an Upside-Down VE

In Experiments 1A and 1B, it appeared that angular expansion with a gain of 1.5 was lessened when the observers made judgments in an empty visual field below an inverted ground plane, but not when there was not a ground plane at all. Moreover, even elevation judgments made upwards toward the inverted ground plane showed a gain of about 1.5. Could the reduced gain in the empty field in this case be due to an artifact of the visual context containing large disparities only in the upper field? Experiment 1C was run to directly compare the estimates of elevation while manipulating the orientation of a more richly structured environment used previously in our lab [59], as shown above in Figure 3a,b. Most notably, this environment provides a distant texture in the sky (simulated at a 3000 m distance—effectively infinity) which allows for the test ball to be viewed without diplopia for farther ball distances. In this experiment, rather than keeping the simulated ball along an arc of a 1 m radius, it was placed (whether up or down) as if along a ground plane (even when in the sky portion of the VE), including appropriate disparities, and projected a smaller visual angle corresponding to its visual distance.

Additionally, the straight-ahead reference ball was at a distance of 50 m, atop a pole embedded into the visible ground (whether inverted or upright), to support the perception of its distance. This was a configuration that had been used previously only for balls placed either along the ground (not the sky) or on an invisible ground, but with a visible horizon line [59], and had been shown to produce an angular gain of about 1.5 in an upright-world, ball-on-ground condition, but with a large positive intercept. Does such a pattern occur when the balls are placed in the sky? Does it occur when the VE is upside down?

**Figure 7:**
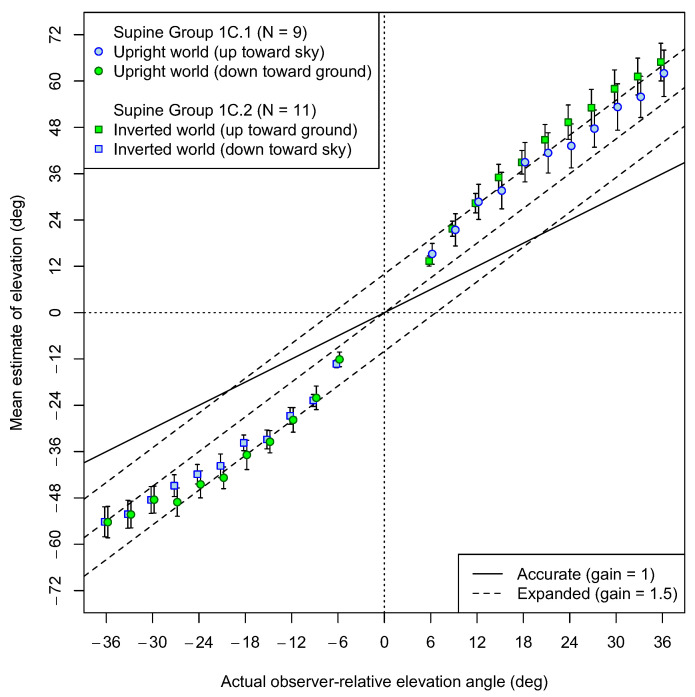
Experiment 1C results (supine participants): Mean estimates of angular elevation both upward (positive) and downward (negative) in each of the two VEs. Square symbols represent estimates given by participants for whom an upside-down ground plane VE was present during estimation. Circular symbols represent estimates made by participants with a normal, upright ground plane in their VE. The colors of the plot points indicate whether the test ball was seen against the sky (blue) or the ground (green). The dashed lines show the prediction of angular expansion by 1.5, as well as that prediction with intercepts of −10° or +10°. The solid line shows where accurate estimates would have fallen. Error bars represent standard errors of the means. Plot points have been offset horizontally by ±0.2 degrees to avoid overlap of error bars.

Figure 7 shows the results of Experiment 1C. It is evident that, under these experimental conditions, the patterns of the results are similar for upright and inverted worlds. Both for upright worlds and inverted worlds, the angular comparison to the distant reference ball seems to add an intercept both for upward and downward estimates of elevation. A previous study described and investigated this pattern of response (though only in the lower visual field) and demonstrated that it was due to the large depth (ground) distance between the reference ball and the test balls [59]. Because of the obvious curvature of the lines, angular gains are not ideal summaries of the data, but the best-fitting slopes in the upper visual field averaged 1.57, which did not differ reliably from 1.5 (*t*(19) = 0.75, *p* = 0.43). This replicates a previous study [59] which showed similar evidence of a 1.5 slope, but with a large intercept when the reference object was at a far distance, as in the present experiment. Similar observations have been made previously when targets were at large distances [61].

The data do not appear to differ between the two orientations of the VE. However, in both VEs, there seems to be a greater curvature for estimates in the lower visual field than in the upper visual field. That is, large negative elevations relative to the observer’s body show a return to an angular gain of 1.5 in both VEs, resulting in mean slopes in the lower visual field (M = 1.29) that are less than 1.5 (*t*(19) = 2.77, *p* = 0.012). Recall that this lower visual field was also the location where there were departures from a strong gain in the inverted VE conditions of Experiments 1A and 1B. It is possible that these effects were caused by looking down toward one’s feet while lying in a supine position, resulting in a cue conflict when the ground plane was inverted (e.g., in Experiments 1A and 1B), but they did not seem to occur in Experiments 1A or 1B when the VE was all sky.

### 3.3. Results of Experiment 2: Convergent Evidence with Upright Observers

So far, then, we have seen strong evidence of angular expansion in perceived elevation across many different contexts, including for supine observers judging elevation in a VE either without a horizon at all, or with an inverted world. Experiment 2 sought to replicate the persistence of angular expansion in the absence of a horizon, even when the participants were sitting upright.

Several techniques have been used to rule out verbal bias in previous studies. One such technique is the psychophysical method of comparison to an internal standard, such as judging whether a slope appears closer to being vertical or horizontal, as a way to measure the perceived 45° point [62,63]. In phase 1, we used this technique to measure the perceived 30° upward elevation point in either an upright ground VE or an (empty) VE with only a sky (we did not try to use 45°, because the display only extended 50° above and below the observer). In each trial, the participants simply indicated whether they thought the ball was more or less than 30° (one-third of the distance between straight ahead and straight up). There were 12 participants in each VE. Their data were fit with cumulative normal psychometric functions where the point of subjective equality (PSE) is the point of maximum uncertainty (50% likely to say more or less), and the just noticeable difference (JND) is defined as the change in orientation required for the curve to reach a 75% likelihood of saying more. The predicted PSE (actual visual direction perceived as 30°), given a gain of 1.5, would be 20°. One participant had a JND that was outside of the pre-registered range, and a second participant had a PSE that was more than 3 standard deviations from the mean. The remaining 22 participants were included in the psychometric analysis. In both VEs, the mean matches observed did not differ from 20°. In the upright-ground VE, the mean observed match was 20.9° (95% CI: 16.9°, 25.0°), and in the VE with only a sky, the mean observed match was 22.2° (95% CI: 16.8°, 27.5°). These means did not differ reliably from each other (*t*(18.5) = 0.4, *p* = 0.67). The mean JND was 2.1° (95% CI: 1.7°, 2.5°).

**Figure 8:**
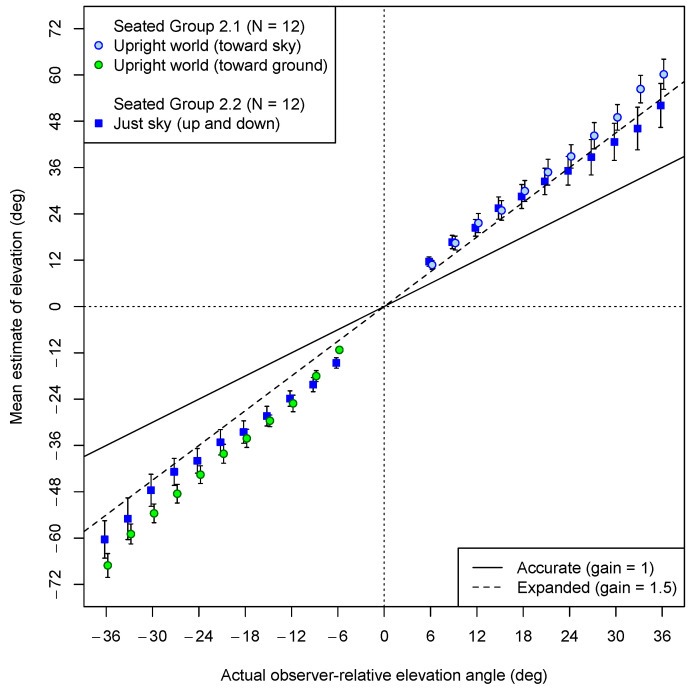
Experiment 2 estimation results (seated participants): Mean estimates of angular elevation both upward (positive) and downward (negative) in each of the two VEs. Blue square symbols represent estimates given by participants in a VE that depicted only the reference ball straight ahead and a single test ball. Circular symbols represent estimates made by participants in a VE with a normal, upright ground plane. The colors of the plot points indicate whether the test ball was against an empty sky (blue) or the ground (green) in the case of the upright VE. The dashed line shows the prediction of angular expansion by 1.5. Error bars represent standard errors of the means. Plot points have been offset horizontally by ±0.2 degrees to avoid overlap of error bars.

Following the psychophysical task in the upper visual field, all participants gave estimates for visual directions both in the lower visual field and then in the upper visual field. The mean estimation data for Experiment 2 are shown in Figure 8.

Overall, the data shown in Figure 8 indicate that the upright observers showed strong angular expansion even when there was not a ground plane in the VE. Separate slopes were computed for upward and downward angles for each participant. A pre-registered ANOVA on the angular gains with VE as a between-participant factor and direction (up or down) as a within-participant factor indicated that the mean gains in the downward direction (M = 1.58, 95% CI: 1.41, 1.77) were reliably larger than the mean gains in the upward direction (M = 1.48, 95% CI: 1.23, 1.67) (*F*(1, 22) = 5.7, *p* = 0.025), although neither mean slope differed reliably from 1.5. The between-participant comparison of slope as a function of VE tested by the ANOVA revealed marginal evidence that the slopes were higher when the ground plane was present (M = 1.68, 95% CI: 1.53, 1.83) than when it was not (M = 1.35, 95% CI: 1.13, 1.58) (*F*(1, 22) = 3.40, *p* = 0.079). There was no evidence of a statistical interaction between the VE and the direction judged (*F*(1, 22) = 0.20, *p* = 0.66).

### 3.4. Results of Experiment 3: No Evidence of Differentiation between Azimuth and Elevation for Supine Observers

The final experiment in this series is reported both for completeness and because its results were unanticipated, but potentially clarifying. The experiment was designed to compare azimuthal gain to elevation gain in supine observers, with azimuthal angles defined as being relative to the head’s orientation in the context of a VE that would either provide an appropriate upright ground surface or not (see Figure 4a above). A previous study found that azimuthal estimates no longer showed significant angular expansion when a blank blue world was used (no horizon) [58], but that was with upright observers. In a gravitational sense, all directions are in elevation when one is lying supine, looking up, but if provided with a visual ground plane in the VE, would supine participants understand angular offsets to the left as azimuth and show a lower gain?

**Figure 9:**
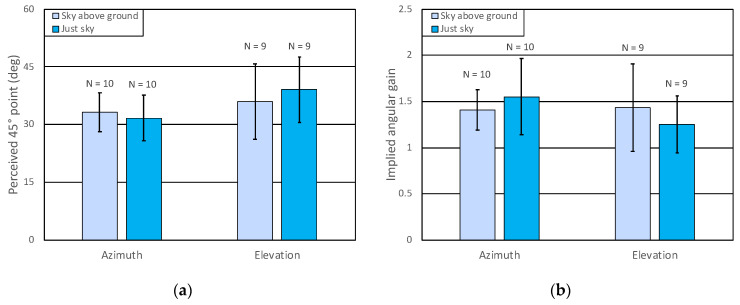
Results of Experiment 3. (**a**) Means of points of subjective equality (perceived 45°) are shown with 95% confidence intervals. (**b**) The means of the implied perceptual gains (45/PSE) are shown as a function of axis and VE with 95% confidence intervals.

Each of the 42 participants saw only one of the two environments and made psychophysical judgments only along one of the two axes. The participants were positioned with their heads near one end of the display, so that the field of view in their upper visual field (for elevation) extended to 66°, or (with the support cart perpendicular to the display) so that the field of view to their left (for azimuth) extended 66°. Their task was to judge whether the direction of the test ball was more than or less than 45° (from the central reference ball) in the relevant axis. After excluding 2 participants with undefined PSEs, 2 more were excluded based on pre-registered criteria concerning the slope of their psychometric functions, leaving 38 participants, with at least 9 in each of the four experimental cells, which met our minimum pre-registered criterion. The pre-registered ANOVA, with the VE and axis as factors, did not find a reliable difference as a function of either variable. Although Figure 9a suggests that the matches were lower for azimuth than for elevation, this effect was not reliable; *F*(1, 34) = 2.45, *p* = 0.126. Nonetheless, the data, as shown in Figure 9a, appear quite different from the pattern that was expected. It is the azimuth condition that appears locked to perceiving a visual direction of about 30° to the left as being 45° (corresponding to perceptual expansion by 1.5). In contrast, the data for elevation seems to show less bias in this case, even though no differences were detected by the planned ANOVA.

Considering that the null result in the ANOVA might be due to the very high variability in the elevation condition, a post hoc test of relative variance confirmed that variability in the elevation condition was marginally higher (2.4 times greater) than that in the azimuth condition; *F*(17, 19) = 2.40, *p* = 0.068.

One possible reason for the increased variability and higher match point for elevation is that we asked the participants in all conditions to respond to stimuli that were presented at visual deviation as much as about 60° relative to the visual direction that was straight ahead (a much greater deviation than those presented in our prior experiments). As a result, some participants in the elevation conditions reported that they needed to tilt their heads back in order to see some of the very highest stimuli. The elevation data from Experiment 3 may therefore be somewhat difficult to interpret for this reason. Head tilt could have encouraged switching reference frames, for example.

Conversely, although the data in the azimuth condition defied the expectations we had in designing the study (that expansion in azimuth would disappear in the absence of a visible ground plane), these data are clearly well behaved. Because peripheral vision has a much larger field of view laterally, the participants would not have had as much difficulty seeing the far lateral stimuli, so the lower variance is unsurprising. But what about the large bias?

In Figure 9b, the data are replotted after converting each participant’s PSE to an implied gain before averaging. Overall, in the azimuth condition, we still see a perceptual gain factor that is very nearly 1.5 with or without a ground plane present, but this is now matched in at least one of the elevation conditions. Notice that in this representation of the data, the variability difference observed for the arithmetic matches no longer holds. Given this outcome for what was meant to be an azimuthal judgment, it is tempting to interpret the results of the azimuth manipulation as evidence of the pattern normally found for elevation.

## 4. Discussion

The series of experiments presented here primarily considered the perception of visual direction in elevation (defined with respect to the body) for supine observers. In most of our virtual environments, including highly reduced (“just sky”) ones, there was evidence of an angular perceptual gain of about 1.5, as has previously been reported in normal outdoor scenes [14,26] and also for upright observers with virtual environments [59]. This is not to say that the environment did not matter. When a far reference ball was used in a rich VE (Experiment 1C), the angular estimates, though their slopes were similar to 1.5, showed a large intercept, so that they greatly exceeded the plot line representing a simple 1.5 gain—just as they can in the real world when a distant reference is used [57,61]. Thus, in relatively rich environments that supported distant depth perception, our supine observers showed effects similar to those found previously for upright observers in the real world with respect to the judgements of angular elevation.

Removing all evidence of a ground plane did little to mitigate the perceptual overestimation of the angular direction. Although other work has suggested that judgments in azimuth can be restored nearly to accurate levels when a ground plane is not present, in none of the conditions tested here, whether with supine observers or with upright observers, did we see angular expansion in elevation entirely disappear as a result of a simplified environment without a ground plane or horizon marker. The exceptional case might be the highly variable elevation data in Experiment 3, but even those data do not provide statistical evidence against expansion by 1.5. Most importantly, it appears possible that supine observers may have treated even lateral directions as if they were elevation angles in Experiment 3.

Thus, the prevalence and robustness of angular expansion in gravitational elevation may be said to be highlighted by the azimuth condition in Experiment 3. In that experiment, using non-numerical responses, and including conditions with neither a ground plane nor any significant environmental structure, the supine participants seemed to behave as if real-world gravity was controlling their judgments. That is, the nominal condition of azimuth (relative to the observer-centric reference frame) appears to have been treated, if anything, as an example of elevation. This is hardly an error, since all deviations from straight up (which is where a supine observer is oriented) are deviations in elevation in the gravitational framework. However, future work should seek to directly compare elevation and azimuth by testing for the large-scale horizontal–vertical illusions in supine participants looking up.

Our present apparatus limited us to very large fields of view in only one axis at a time. The results of Experiment 3 suggest that it would be interesting to directly test for a large-scale horizontal–vertical illusion (HVI) with supine observers by having them match the apparent relative lengths of horizontal bars presented in depth that are either parallel with or perpendicular to the sagittal plane of the observer. It is well established that the small-scale HVI (a 5% effect) is retinotopic. Evidence suggests that the larger horizontal–vertical illusion found for large objects (a 20% effect) is not retinotopic [23] when measured directly with sideways observers at a normal eye height (as simulated in the present experiments), though others, measuring one axis at a time from a lower viewing point, have reported size-matching evidence that is consistent with large angular gains in both axes with sideways observers [55]. However, given that large angular gains were found for both azimuth and elevation with supine observers looking up, it seems possible that only the small-scale HVI would emerge in the reduced (ground only or sky only) VEs used for Experiment 3. Alternatively, it could be that the use of the asymmetric field of view is why we observed a similar expansion in azimuth and elevation (which were each tested along the long axis of the apparatus) in Experiment 3, and that this factor would determine the HVI for supine observers.

There are a large number of studies in the literature on interactions between vision and the vestibular system with respect to the perception of orientation [64,65], but also of motion. Previous studies have validated the use of supine observers for studying the perceived heading direction, for example, in the presence of an appropriate optical flow field [66], but have not examined measures of angular gain. Other studies have looked at perceived (frontal) orientation relative to one’s own body along different axes and found little orientation bias for supine observers looking upward, but found clear interactions among vestibular, visual, and somatic information for side-gazing supine observers [67,68]. The present studies have sought to contribute to the body of literature by bringing a ground-based perspective [69,70] to the study of supine observers looking up and judging visual direction in elevation. In the absence of visual information specifying an alternative reference frame, the data are fairly consistent in showing a 1.5 gain in perceived elevation relative to straight-ahead. Anomalies in some conditions might be attributed to participant awareness of alternative or conflicting reference frames.

The present results, in combination with what we already know about real-world judgments and behavior, seem to re-affirm that participants’ behaviors, with respect to the perception of elevation, tend to reflect an allocentric reference frame defined by the gravitational framework. Although rich, immersive visual information can influence the choice of reference frame [23], the default framework for the angular expansion of perceived elevation may be gravitational, even for supine observers.

## Figures and Tables

**Table 1:** Designs of magnitude estimation in Experiments 1A, 1B, 1C, and 2 on perceived elevation.

Experiment	Manipulated Between Ss	Manipulated Within Ss ^1^
1A (supine)	Orientation of environment (upside down or normal)	1. Presence of ground 2. Direction of deviation (blocked)
1B (supine)	Presence of ground (always upside down)	Direction of deviation (not blocked)
1C (supine)	Orientation of environment ^2^ (upside down or normal)	Direction of deviation (blocked)
2 (seated)	Presence of ground (always right side up)	Direction of deviation (blocked)

^1^ Angular deviation magnitude also varied within subjects in all three of these experiments. ^2^ A richer environment was used here, with a far reference ball and a varied-distance test ball.

**Table 2:** Designs of internal standard psychophysical methods in Experiments 2 (phase 1) and 3.

Experiment	Manipulated between Ss	Internal (Subjective) Standard
2 (seated)	Presence of (upright) ground	Upward 30° elevation
3 (supine)	1. Presence of (upright) ground 2. Axis (elevation or azimuth)	Upward 45° in elevation or Leftward 45° in azimuth

## Data Availability

Complete data and R analysis files will be made available at OSF (https://osf.io/a2xue/?view_only=08e3dfaafcde47a0b930d33d800327bc (accessed on 15 February 2024)).
